# Glycaemic Control after Metformin Discontinuation in Diabetic Patients with a Declining Renal Function

**DOI:** 10.1155/2017/2769819

**Published:** 2017-11-05

**Authors:** Theresa Leyco, Davin Ryanputra, Ray Peh, Alexphil Ponce, Chin Meng Khoo

**Affiliations:** ^1^Department of Medicine, National University Hospital, Singapore; ^2^Yong Loo Lin School of Medicine, National University of Singapore, Singapore; ^3^Higher Education Department, Centre for International Education, Cebu, Philippines

## Abstract

Metformin is contraindicated in diabetic patients with declining renal function. This study examined the glycaemic control in diabetic patients with chronic kidney disease when metformin was discontinued. This was a retrospective study. We screened 2032 diabetic patients who attended the Diabetes Clinic at a tertiary hospital between 1 September 2014 and 30 September 2015. We analyzed the data on 69 patients whom metformin was discontinued due to declining renal function and had a complete 6-month follow-up. There was no significant difference in the HbA1c and body weight at 6-month follow-up compared to baseline after metformin discontinuation. The eGFR was significantly lower at 6-month follow-up compared to baseline. Upon metformin discontinuation, the majority of patients had their diabetes medication uptitrated (in particular insulin or sulphonylurea). Patients with an improved glycaemia at 6-month follow-up had further declined in eGFR compared to patients with worsened glycaemia. 17% of the study patients experienced hypoglycaemia. Upon metformin discontinuation, glycaemic control could be optimised with uptitration but should be balanced against the risk of hypoglycaemia. Further improvement in the glycaemic control might indicate further deterioration in the renal function.

## 1. Introduction

The rising prevalence of type 2 diabetes mellitus (T2DM) is considered one of the most challenging public health problems. More than 400 million people will be affected by T2DM by the year 2030, with the greatest increase expected in Asian populations [1, 2]. Metformin is the recommended first-line antidiabetic therapy for all patients in addition to lifestyle change. Metformin is a very effective antidiabetic agent, widely available and affordable. Its most common side effect is gastrointestinal irritation. T2DM is associated with dismal micro- and macrovascular complications. One of the most dreadful microvascular complications is diabetic nephropathy, which is often characterised by declining estimated glomerular filtration rate (eGFR) and proteinuria. Declining renal function prohibits many medications for fear of potential side effects from lower renal clearance. As such, it is recommended that metformin should be discontinued when the eGFR falls to 30 ml/minute/1.73 m^2^ or below [3], in anticipation of a higher risk of lactic acidosis. Other antidiabetic agents including sulfonylureas, meglitinides, dipeptidyl peptidase-4 inhibitors, and insulin have been shown to be safe in patients with declining renal function [4].

This study aimed to examine the effect of metformin discontinuation on glycaemic control among diabetic patients with declining renal function. We hypothesised that the glycaemic control would worsen with metformin discontinuation. We also examined the various treatment strategies employed in a real-world practice to mitigate the worsening of glycaemic control upon metformin discontinuation.

## 2. Materials and Methods

### 2.1. Study Design and Subjects

This is a retrospective study conducted in the Diabetes Clinic, National University Hospital, Singapore. Data on all adult patients with T2DM (aged 21 years and above) who had attended the diabetes clinic between 1 September 2014 and 30 September 2015 were screened for metformin withdrawal. We selected patients whom metformin was withdrawn due to worsening renal function and had a complete 6 months of follow-up after metformin discontinuation. Patients were excluded if they fulfilled any of the following criteria during the period when metformin was withdrawn: were on erythropoietin (EPO) therapy, received steroid therapy for at least 1 week, had an average haemoglobin level of less than 10 g/dL, had episode(s) of active bleeding or received packed red cell transfusion, or received renal replacement therapy.

### 2.2. Data Collection

This study was approved by Singapore's National Healthcare Group Domain Specific Review Board (DSRB Ref number C2016/00283). Data collected included age, sex, ethnicity, duration of diabetes, weight in kg, height in m, blood pressure (BP) in mmHg, average haemoglobin level, lipid profile, glycosylated haemoglobin (HbA1c) concentration, and serum creatinine. HbA1c concentration was measured using the Siemens/Bayer DCA 2000+ Analyser point-of-care management platform. The estimated glomerular filtration rate (eGFR) was calculated using the CKD-EPI equation. Data on hypertension, hyperlipidemia, ischemic heart disease (IHD), cerebrovascular disease (CVD), peripheral vascular disease (PVD), diabetic retinopathy, diabetic neuropathy, and medication history were also obtained. Hypoglycaemic episodes were recorded based on available self-monitoring blood glucose data or based on a physician's assessment during the clinic visit. All the data were collected at the clinical visit when metformin was withdrawn (baseline) and at 3 and 6 months after metformin withdrawal (follow-up). In contrast to the available recommendation to discontinue metformin, there were no guidelines on the management plan upon metformin discontinuation. Thus, diabetes management following metformin discontinuation was at the discretion of the physician-in-charge.

### 2.3. Statistical Analysis

Data analysis was performed using Minitab (version 16 for Windows; Minitab Inc., State College, PA) and SPSS (version 16 for Windows; SPSS, Inc., Chicago, IL). Values are expressed as means [standard deviation (SD)] unless stated otherwise. The paired *t*-test was used to examine the changes in HbA1c at 3 and 6 months from baseline. A repeated measure ANOVA was used to examine the change in HbA1c, body weight, and eGFR throughout the 6-month follow-up following metformin discontinuation. Wilcoxon signed-rank and Mann-Whitney *U* tests were used to examine the change in the diabetes treatment regime at baseline and at 3 months of follow-up. A *p* value <0.05 was considered statistically significant.

## 3. Results

**Figure 1:**
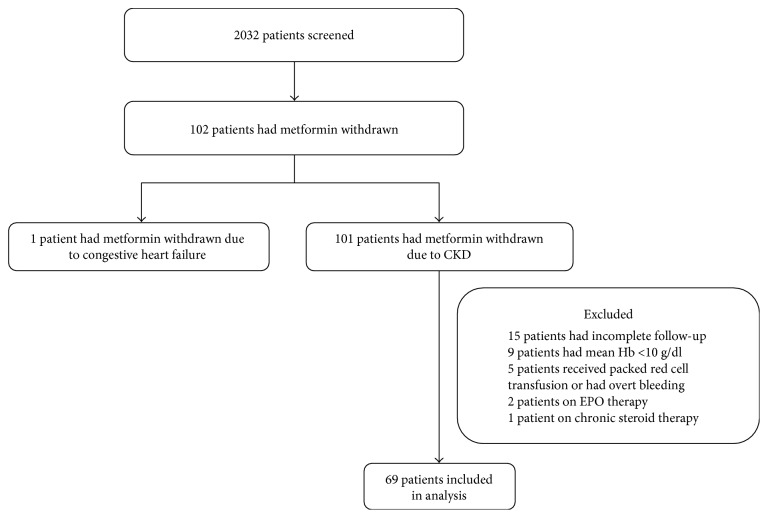
Recruitment of study population.

A total of 2032 patients who attended the Diabetes Clinic, National University Hospital, Singapore between 1 September 2014 to 30 September 2015 were screened (Figure 1). There were 102 patients (5.02%) who had metformin discontinued. One patient had metformin discontinued due to congestive heart failure and was excluded from the analysis. The remaining 101 (4.97%) had metformin discontinued due to worsening renal function. Of these, 15 patients were excluded for incomplete follow-up data, nine patients for haemoglobin (Hb) concentration less than 10 g/dl, five patients for having received transfusion of packed red cells or had overt bleeding during the study period, two patients for having received EPO injection, and one patient for being on chronic steroid therapy.

Baseline patients' characteristics are shown in Table 1. Relevant to this study, the mean serum creatinine (SD) was 186.1 *μ*mol/L (56.9 *μ*mol/L) with the corresponding mean eGFR was 29.7 ml/min/1.73 m^2^ (13.0 ml/min/1.73 m^2^) at metformin discontinuation. Twenty-eight patients (40%) had metformin discontinued even when their baseline eGFR was between 30 and 60 ml/min/1.73 m^2^. Body weight did not change significantly throughout the 6-month study period (*p* = 0.148). However, the mean eGFR declined significantly from 29.7 ml/min/1.73 m^2^ (13.7 ml/min/1.73 m^2^) to 26.4 ml/min/1.73 m^2^ (11.2 ml/min/1.73 m^2^) (*p* = 0.049). The mean HbA1c was 8.8% (2.3%) at baseline, 9.2% (2.2%) at three months, and 9.0% (2.2%) at 6-month follow-up (*p* = 0.262).

We identified two groups that had improved (*n* = 25, HbA1c decreased >0.5% at 6-month follow-up from baseline) and worsened glycaemic control (*n* = 25, HbA1c increased >0.5% at 6-month follow-up from baseline) (Table 2). There were 19 patients whose HbA1c change was less than 0.5% during the 6-month follow-up. Compared to the improved glycaemia group, worsened glycaemia group had a significantly longer duration of diabetes but lower mean HbA1c at baseline. Majority of the patients in both groups were on insulin therapy. There was no significant difference between groups in the use of oral antidiabetic agents at baseline. The eGFR remained stable in the group with worsened glycaemia but further declined significantly in the group with improved glycaemia.

**Figure 2:**
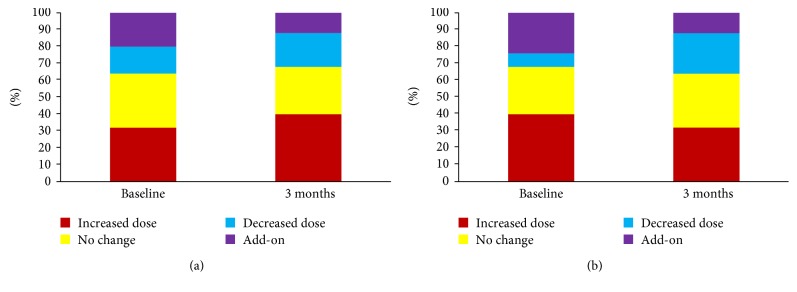
(a) Changes in diabetes treatment regime following metformin discontinuation in group with worsened glycaemia (HbA1c increased >0.5% at the 6-month follow-up from baseline). *p* < 0.05 comparing proportion of increased dose between 3 months and baseline. (b) Changes in diabetes treatment regime following metformin discontinuation in group with improved glycaemia (HbA1c decreased >0.5% at 6-month follow-up from baseline). *p* < 0.05 comparing proportion of increased dose or decreased dose between 3 months and baseline.

Figures 2(a) and 2(b) show the changes in the diabetes treatment regime following metformin discontinuation. Upon discontinuation of metformin, the majority of the patients in the improved and worsened glycaemia group had their medication uptitrated or add-on another medication (in particular DPP-IV inhibitor). Compared to baseline, a large proportion of patients in the worsened glycaemia group had uptitration in medication dose (in particular insulin) (*p* < 0.001) at 3 months of follow-up after metformin discontinuation. In the improved glycaemia group, there were significant differences in the decrease in the proportion of patients with uptitration in medication (*p* < 0.001) and in the increase in the proportion of patients with downtitration of medication (*p* = 0.046) at 3 months of follow-up compared to baseline.

Twelve out of the 69 patients (17%; 5 from worsened glycaemia group, 5 from improved glycaemia group, and 2 with a HbA1c change of less than 0.5% in 6 months) reported hypoglycaemic episodes. All patients were on insulin therapy.

## 4. Discussion

We reported the changes in the glycaemia level and glycaemia management among diabetic patients in whom metformin was discontinued due to declining renal function. It is recommended that metformin should be discontinued once eGFR falls below 30 ml/min/1.73 m^2^ and to decrease the metformin dose in mild to moderate renal impairment (eGFR 30–60 ml/min/1.73 m^2^). We noted that 40% of the study subjects had metformin prematurely withdrawn when eGFR was between 30 and 60 ml/min/1.73 m^2^, and therefore they were potentially deprived of the overall reduction in morbidity and mortality, low hypoglycaemia risk, and cardiovascular benefits of metformin [5, 6].

While there is a clear recommendation on metformin discontinuation when renal function declines, there is no recommendation on how to manage the glycaemia upon metformin discontinuation. We found that there was no significant change in the glycaemic control at 6-month follow-up after metformin discontinuation. Majority of the patients had their medication uptitrated or had an additional antidiabetic agent when metformin was discontinued.

Although we did not find a significant change in the glycaemic control 6 months after metformin discontinuation, we observed that the glycaemic control deteriorated in the first 3 months after metformin discontinuation. Interestingly, we found that there were two groups of patients that experienced worsening or improvement in the glycaemic control at 6-month follow-up. The worsening of glycaemia was expected after metformin discontinuation, but optimisation of antidiabetic agents at metformin discontinuation and 3 months of follow-up did not stem off the worsening of glycaemia at 6-month follow-up in the group with worsened glycaemia. Similar treatment optimisation was observed in the group with improved glycaemia, and in fact, the treatment regimen was downtitrated at 3-month follow-up. A further decline in the renal function in the improved glycaemia group likely contributes to the improvement in glycaemia and explains the downtitration of treatment regimen at 3-month follow-up.

Glucose homoeostasis is complex in diabetic nephropathy. Insulin resistance plays a key role in the worsening of glycaemic control in patients with a decline renal function [7–9]. The increased insulin resistance is the effect of accumulated uremic toxins and increased oxidative stress [10]. Among the many antiglycaemic actions of metformin is an improvement in insulin sensitivity directly by increasing insulin tyrosine kinase receptor activity and indirectly by reducing lipotoxicity [11]. Thus, discontinuation of metformin in diabetic individuals will result in greater insulin resistance and worsening glycaemic control. Beside metformin and PPAR-gamma agonist, there is no other antidiabetic agent that would address the pathophysiologic insulin resistance. Thus, increasing the dose of insulin or sulphonylurea or the addition of a DPP-IV inhibitor might not be so effective in controlling glycaemia after metformin discontinuation. This was evident in the group with worsening glycaemia after metformin discontinuation in this study. However, consistent with the current opinion, improvement in the glycaemia in patients with diabetic kidney disease might suggest a further decline in renal function, and this is observed in our study. The decrease in the renal capacity of gluconeogenesis reduced clearance of antidiabetic agents, and poor nutrition might contribute to the improvement in glycaemic control or recurrent hypoglycaemia in patients with declining renal function [12–15].

Importantly, the optimisation of antidiabetic treatment must take into consideration the risk of hypoglycaemia. About 20% of our patients experienced hypoglycaemia within 6 months of optimisation of antidiabetic treatment. Aside from poor renal function, other risk factors for hypoglycaemia were seen in these patients such as old age, long duration of diabetes, high glycosylated haemoglobin, and the use of insulin. In the group with improved glycaemia, there was less treatment intensification at 3 months of follow-up, and this might have mitigated the hypoglycaemia risk in this group.

This study has several limitations. It is a retrospective study. We could not validate compliance to medications and dietary restriction. Our study has a small sample size, and this is due to our strict inclusion and exclusion criteria. We did not take into consideration patients whom metformin dose was reduced given the early decline in renal function (eGFR between 30 and 60 ml/minute/1.73 m^2^). None of our patients were on PPAR-agonist or GLP-1 agonists. Thus, our interpretation of the data is limited to increasing the dose or addition of insulin, sulphonylurea, or DPP-IV inhibitor upon metformin discontinuation.

In conclusion, upitration of existing insulin or sulphonylurea or addition of DPP-IV inhibitor might help to mitigate deterioration in glycaemic control upon metformin discontinuation due to declining renal function. In patients with continuous improvement in glycaemic control, it is important to follow the renal function closely, as this may be a harbinger of a rapid decline in renal function toward end-stage kidney disease. Also, the optimisation of diabetes treatment should take into consideration the risk of hypoglycaemia in patients with a declining renal function.

## Figures and Tables

**Table 1:** Study patients characteristics.

	*N*	(%)	Mean	SD
Age, years			66.7	10.9
40–49	3	4		
50–59	19	28		
60–69	19	28		
70–79	20	29		
80–89	7	10		
90–99	1	2		
Male	37	54		
Ethnicity				
Chinese	37	54		
Malay	28	41		
Asian-Indian	4	6		
Diabetes duration, years			18.2	8.3
HbA1c, %			8.8	2.3
Weight, kg			71.8	12.9
Height, m			1.56	0.1
Body mass index, kg/m^2^			28.0	5.8
Hypertension	65	94		
Hyperlipidemia	62	90		
Total cholesterol, mmol/L			4.9	19.6
Triglyceride, mmol/L			2.2	1.4
HDL, mmol/L			1.2	0.3
LDL, mmol/L			2.7	1.2
Ischemic heart disease	40	58		
Cardiovascular disease	12	17		
Peripheral vascular disease	12	17		
Diabetic retinopathy	22	32		
Diabetic neuropathy	7	10		
Serum creatinine, *μ*mol/L			186.1	56.9
eGFR, ml/min/1.73 m^2^			29.7	13.0
Hemoglobin, g/dl			11.8	1.4

**Table 2:** Characteristics of the patients with improved glycaemia (HbA1c decreased >0.5% at 6-month follow-up from baseline) and worsened glycaemia (HbA1c increased >0.5% at 6-month follow-up from baseline) following metformin discontinuation.

	Total cohort (*n* = 69)	Worsened glycaemia (*n* = 25)	Improved glycaemia (*n* = 25)	*p* value comparing worsened versus improved glycaemia
Age, years	66.7 (10.9)	68.1 (9.13)	65.0 (13.3)	0.339
Male, %	54	60	64	0.771
Diabetes duration, years	18.2 (8.3)	20.4 (7.48)	15.3 (7.4)	**0.020**
Medications at metformin discontinuation, %				
Insulin	51	52	56	0.776
Sulfonylurea	42	40	36	0.771
DPP-IV inhibitor	7	8	8	1.000
HbA1c, %				
At baseline	8.8 (2.3)	8.1 (2.0)	10.1 (2.5)	**0.002**
At 3 months	9.2 (2.2)	9.8 (2.6)^a^	9.1 (2.3)	
At 6 months	9.0 (2.2)	10.4 (2.6)^b^	8.3 (1.7)^b^	
Body weight, kg				
At baseline	71.8 (12.9)	75.3 (14.4)	68.7 (14.4)	0.107
At 3 months	72.4 (13.0)	75.5 (13.9)	71.4 (13.6)	
At 6 months	72.8 (12.2)	76.4 (13.1)	71.1 (14.0)	
eGFR, ml/minute/1.73 m^2^				
eGFR at baseline	29.7 (13.0)	29.6 (8.5)	33.0 (12.3)	0.250
eGFR at 3 months	27.5 (10.8)	30.1 (14.7)	25.9 (10.5)^a^	
eGFR at 6 months	26.3 (11.2)	29.8 (17.2)	23.8 (9.1)^b^	

Values are in mean (SD) unless stated otherwise. ^a^*p* < 0.05 for comparison between baseline and at 3-month follow-up. ^b^*p* < 0.05 for comparison between baseline and at 6-month follow-up.
